# Immunomodulatory Effects of an Aqueous Extract of Black Radish on Mouse Macrophages via the TLR2/4-Mediated Signaling Pathway

**DOI:** 10.3390/ph15111376

**Published:** 2022-11-09

**Authors:** Hyungsik Jeon, Soyeon Oh, Eunjoo Kum, Sooyeong Seo, Youngjun Park, Giok Kim

**Affiliations:** 1 Biodiversity Research Institute, Jeju Technopark, Seogwipo 63608, Korea; 2 Yuyu Healthcare Inc., 59-11. Ucheonsaneopdanji-ro, Ucheon-myeon, Heengseong-gun 25244, Korea; 3 Jeju Research Institute of Pharmaceutical, College of Pharmacy, Jeju National University, Jeju 63243, Korea

**Keywords:** black radish, mouse macrophage, immunostimulatory, toll like recepter, nitric acid(NO), ROS, phagocytosis, cytokine

## Abstract

Here, we determined the immunostimulatory effects of black radish (*Raphanus sativus* ver niger) hot water extract (BRHE) on a mouse macrophage cell line (RAW 264.7) and mouse peritoneal macrophages. We found that BRHE treatment increased cell proliferation, phagocytic activity, nitric oxide (NO) levels, cytokine production, and reactive oxygen species synthesis. Moreover, BRHE increased the expression of the following immunomodulators in RAW 264.7 cells and peritoneal macrophages: pro-inflammatory cytokines (IL-1β, IL-6, and TNF-α), iNOS, and COX-2. BRHE treatment significantly up-regulated the phosphorylation of components of the mitogen-activated protein kinase (MAPK), nuclear factor-κB (NF-κB), Akt, and STAT3 signaling pathways. Further, the effects of BRHE on macrophages were significantly diminished after the cells were treated with the TLR2 antagonist C29 or the TLR4 antagonist TAK-242. Therefore, BRHE-induced immunostimulatory phenotypes in mouse macrophages were reversed by multiple inhibitors, such as TLR antagonist, MAPK inhibitor, and Akt inhibitor indicating that BRHE induced macrophage activation through the TLR2/4–MAPK–NFκB–Akt–STAT3 signaling pathway. These results indicate that BRHE may serve as a potential immunomodulatory factor or functional food and provide the scientific basis for the comprehensive utilization and evaluation of black radish in future applications.

## 1. Introduction

Polysaccharides derived from natural resources, such as plants, animals, bacteria, fungi, algae, and arthropods serve as metabolic modulators and structural components required for numerous and diverse biological functions. Such functions include signal recognition, intercellular connection, immune system regulation, and blood coagulation. Further, polysaccharides serve as therapeutics [1,2,3]. Notably, polysaccharide-containing substances are used as wound dressings, drug delivery systems, scaffolds, and coatings for tissue-engineering [2]. The high bioavailability and natural abundance of polysaccharides contribute to their use in numerous applications [4]. Moreover, evidence indicates the ability of plant polysaccharides to modulate the immune system without inducing adverse effects [5], leading to the conclusion that most bioactivities of polysaccharides represent their indirect effect on immunomodulation [6]. The physicochemical and biological properties of plant-derived polysaccharides include immunomodulation [7], anticancer activity [8], neuroprotection [9], antioxidant effects [10], and prebiotic functions [11].

Macrophages stimulated with polysaccharides are activated by MAPK family components such as ERK, JNK, and p38 through the Toll-like receptor (TLR) cellular signaling pathway. Polysaccharide-induced stimulation and activation of MAPK in association with TLRs mediate the generation of inflammatory cytokines, nitric oxide (NO), and reactive oxygen species (ROS) [12,13]. Further, macrophages are directly or indirectly activated through the NFκB, PI3K/Akt, and STATs signal transduction pathways in response to invading pathogens, foreign substances, and immunomodulators, respectively. Natural immunomodulators include mushrooms [14], ellagic acid [15], ginseng [16], *Aloe vera* [17], and *Astragalus* [18].

Radish has been used in folk medicine since ancient times as a laxative, stimulant, digestive aid, and appetite stimulant as well as a drug to treat urinary infections, hepatic inflammation, cardiac disorders, ulcers, and stomach disorders [3,19,20]. Radishes comprise a rich complement of functional components, such as glucosinolates, flavonoids, organic acids, anthocyanin, polyphenol, and minerals, which help in the treatment of numerous diseases [21,22,23].

Recent reviews describe the therapeutic activities of polysaccharides isolated from white radish roots and leaves, many of which contribute to the regulation of the immune response. For example, Son et al., summarize the structural characteristics and immunostimulating properties of polysaccharides from radish leaves, including those with antimetastatic activity and NK cell activity [24]. Inaba et al. investigated the structure of polysaccharides released from the carbohydrate moieties of a radish leaf [25]; Du et al. (2018) discussed an analytical method that distinguishes N-glycan structures of different radish cultivars [26]; Schafet et al. (2016) characterized the cell well composition of the radish root [27]; and Kim et al. (2021) conducted a detailed review of radish sprout that alleviated disease in a mouse model of DSS [28]. Here, we determined the immunomodulatory activities of polysaccharides of the black radish, including their chemical compositions, functions, and the mechanisms of their immunoregulatory effects.

Recent research shows that plant polysaccharides partially exert their immunomodulatory effects through the TLR2,4/NFκB signal transduction pathway [18,29,30]. However, the mechanism underlying the immunostimulatory effects mediated by TLR4 and whether other TLR receptors and signaling mechanisms are involved remains to be determined. Further, numerous published studies that directly compare immunomodulators do not include analyses of the immunomodulatory effects of radish polysaccharides.

Here, we aimed to determine whether radish polysaccharides and their metabolites exert immunomodulatory effects through TLRs because, as mentioned above, few reports have analyzed the immunomodulatory effects and mechanisms of action of BRHE on the mouse macrophage cell line RAW 264.7 and on mouse peritoneal macrophages. To the best of our knowledge, the present study is the first to investigate the role of the TLR2/4–MAPK–NFκB–Akt–STAT3 signal transduction pathway in mediating the immunomodulatory effects of BRHE. Here, we (1) evaluate the enhancing effect of BRHE on the secretion of inflammatory cytokines (NO, IL-6, IL-1β, and TNF-α) and the production of immunomodulatory factors (iNOS, Cox-2); (2) evaluate the levels of mRNAs encoding IL-6, IL-1β, TNF-α, inducible nitric oxide synthase (iNOS), and COX-2 in RAW 264.7 cells and peritoneal macrophage; (3) evaluate the effects of ROS and phagocytosis on the immunostimulatory effects of BRHE; (4) determine whether BRHE activates (i.e., phosphorylated) MAPK, NFκB (p65), STAT3, and Akt; and (5) determine whether BRHE activates TLR2/4-triggered signaling and induces immunostimulation through TLR2/4 in the presence of TLR2/4 inhibitors.

## 2. Results

### 2.1. Extract and Molecular Weight of the BRHE

**Figure 1:**
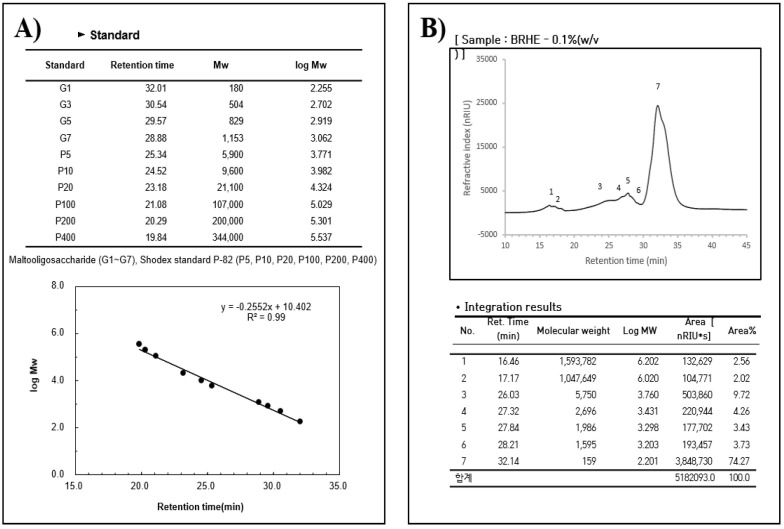
High-performance size exclusion chromatography (GPC-RI)) profiles of BRHE. The calibration curve of standards (the molecular weight of the standard curve) (**A**). Molecular weight distribution of BRHE (**B**). A calibration curve of 10 standard Maltooligosaccharides (G1~G7) and Shodex standard P82 (P5~P400) was used for interpreting the results.

Hot water extraction is an efficient technique for preparing BRHE. The yield of crude BRHE polysaccharides after freeze-drying was 38.7% ± 1.5 (Table 1). The molecular mass range was approximately 158~1,593,782 Daltons according to the standard calibration curve (Figure 1A). The GPC-RI chromatogram of the BRHE showed seven peaks at different elution times (time in min (Daltons)): 16.46 (1,593,782), 17.17 (1,047,649), 26.03 (5750), 27.32 (1986), 28.21 (1595), 32.14 (159) (Figure 1B). 

### 2.2. Analysis of Monosaccharide and Composition

**Figure 2:**
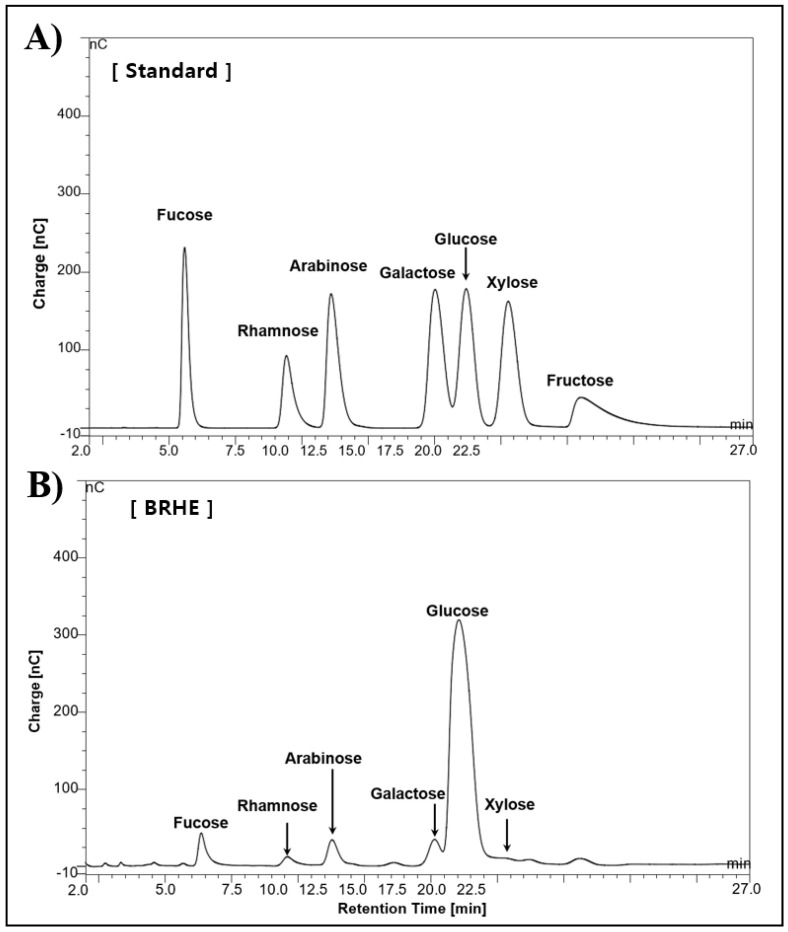
The HPLC chromatograms of seven standard monosaccharides (**A**), and BRHE component monosaccharides of the black radish hot water extracts (**B**), The HPLC analysis was carried out as described in the experimental section. Peaks: (1) Fucose, (2) Rhamnose, (3) Arabinose, (4) Galactose, (5) Glucose, (6) Xylose and (7) Fructose.

BRHE consisted of 71.3% carbohydrate, 9.6% protein, 0.1% fatty acid, 12.4% ash content, and moisture 6.6% (Table 1). Future more, the carbohydrate was composed of 42% sugar, 12% polysaccharide and 2.5% acid sugar. The polysaccharide composition of BRHE is shown in Figure 2A,B. Glucose (167 mg/g), rhamnose (5 mg/g), arabinose (5 mg/g), galactose (5 mg/g), and xylose (n.a) were present in the BRHE at the ratios as follows: 84.5:5.0:5.0:2.0:2.7, respectively. The amount of sugars in the BRHE are shown in Figure 2B. As shown in Figure 2A, seven monosaccharides were detected in the seven polysaccharide fractions as follows: fucose, rhamnose, arabinose, galactose, glucose, xylose, and fructose, and the amount of glucose among the seven monosaccharides was the highest (84%).

### 2.3. Effect of BRHE on Cell Viability and NO Production

**Figure 3:**
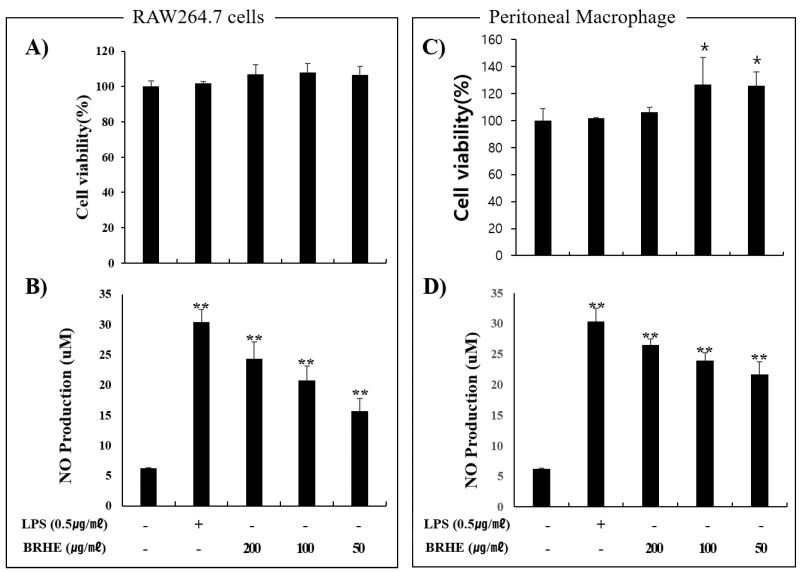
Effects of black radish hot water extract (BRHE) on the cell viability and NO production of RAW 264.7 cells and peritoneal macrophages. The cells were incubated for 20 h with different concentrations of BRHE (200, 100, and 50 μg/mL) with or without 0.5 μg/mL LPS. The cell viability (**A**,**C**) and NO production (**B**,**D**) were measured using WST assay and Griess assay as described in the Material and Methods. Data are expressed as mean ± SD (n = 3) of the three independent experiments. * *p* < 0.05, ** *p* < 0.01 versus the untreated control group.

The BRHE was not cytotoxic to RAW 264.7 cells and PMs, upon BRHE treatment at variable concentrations, the viability of RAW 264.7 cells was comparable to that of BRHE-untreated cells. In PMs, cell viability was slightly increased at 50 and 100 μg/mL of BRHE (Figure 3A,C). Moreover, BRHE tended to increase the proliferation of peritoneal macrophages compared with that of the RAW 264.7 cells (Figure 3C). To evaluate the effect of BRHE on the activation of mouse macrophages, we determined NO production in RAW 264.7 cells (Figure 3B) and PMs (Figure 3D). As shown in Figure 3, 50~200 μg/mL BRHE significantly stimulated the production of NO in RAW 264.7 cells and PMs in a concentration-dependent manner (Figure 3B,D). Notably, the production of NO in the presence of 200 μg/mL BRHE was similar to that of cells treated with 0.5 μg/mL LPS. The increased production of NO suggests that BRHE may activate macrophages.

### 2.4. BRHE Enhances ROS Production

**Figure 4:**
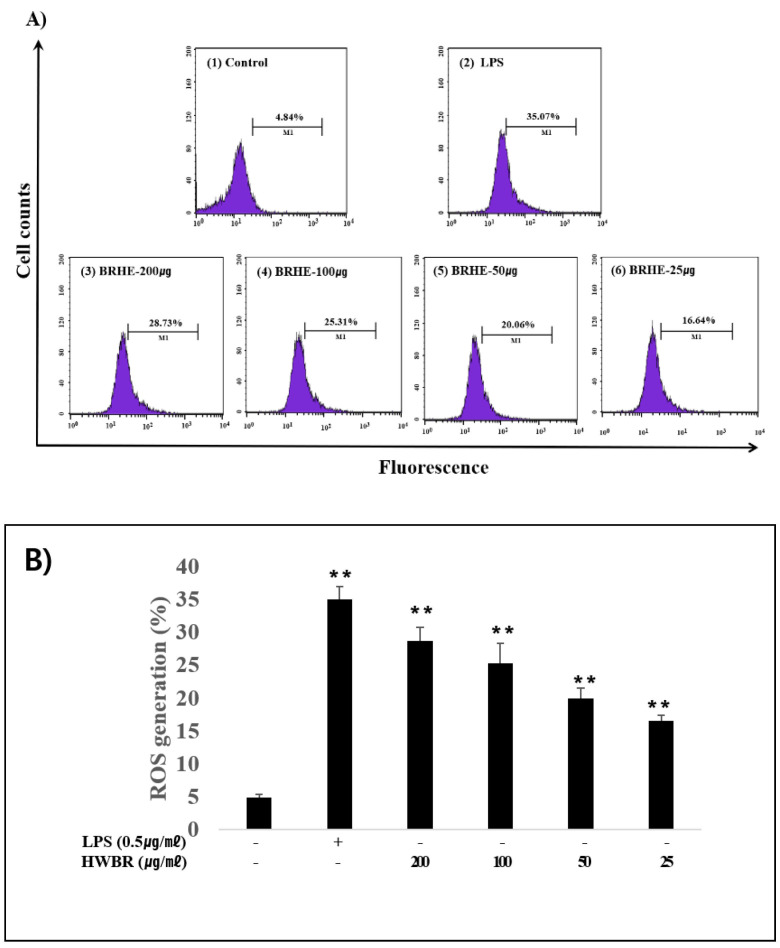
Effect of BRHE on ROS production in RAW 264.7 macrophages. Cells were treated with 200, 100, 50 and 25 μg/mL BRHE or treated with 0.5 μg/mL of LPS for 20 h. (**A**) Representative flow cytometry histogram overlay plots of DCF in RAW 264.7 macrophages. (**B**) Statistical analysis of the mean fluorescence intensity of DCF. Fluorescence microscopic images of cells stained for ROS. Data are shown as the mean ± SD of three replicates (n = 3). ** *p* < 0.01, compared to the control group.

Here, we investigated whether BRHE could affect ROS production, because ROS production can induce autophagy and TLR can influence autophagy activity. The effect of BRHE-induced intracellular ROS production was demonstracted in RAW264.7 cells using DCFH-DA staining and FACS analysis (Figure 4A). Noteable, adding BRHE to final concentrations of 200, 100, 50 and 25ug/ml increased ROS production by 28.73%, 25.31%, 20.06%, and 16.16%, respectively (Figure 4B). BRHE significantly increased intracellular ROS levels in a concentration-dependent manner. These results suggest that BRHE-induce ROS production in macrophages, and that ROS is involved in im-munostimulatory functions through phagocytosis activation [31].

### 2.5. Effect of BRHE on the Phagocytic Activity of RAW 264.7 Cells

**Figure 5:**
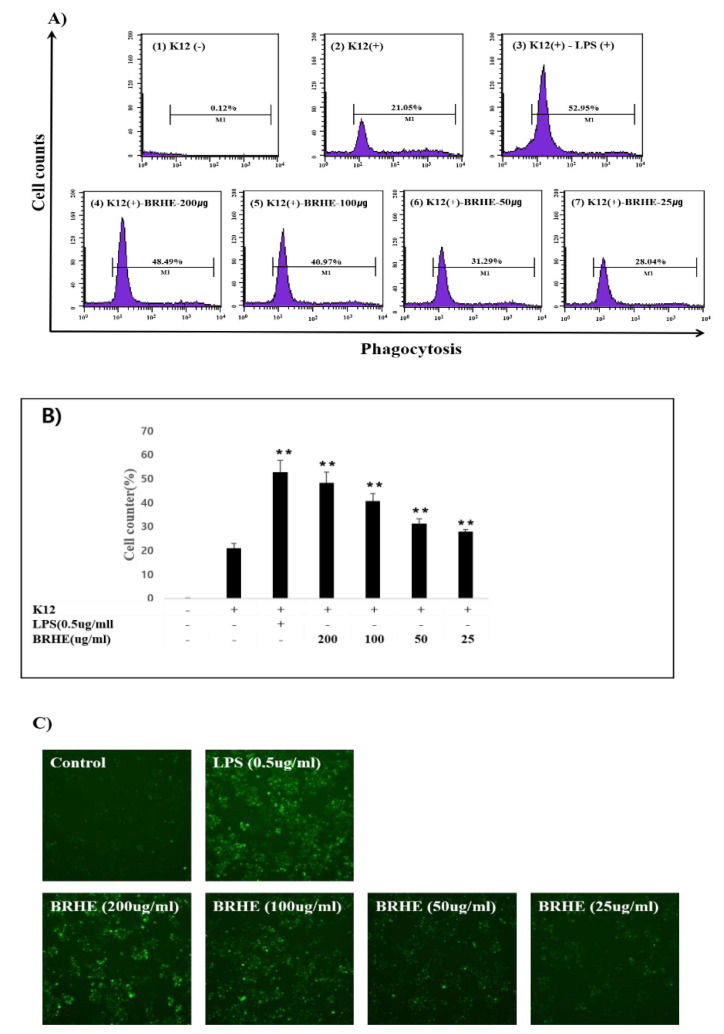
Effect of BRHE on phagocytosis in RAW 264.7 cell line. RAW 264.7 cells were treated with 200, 100, 50, and 25 μg/mL of BRHE or 0.5 μg/mL of LPS in the presence of K12 particles. (**A**) Phagocytosis of K12 particle was analyzed by FACS. (1) vehicle only (control); (2) K12 particle; (3) K12+0.5 μg/mL LPS, (4) K12 –BRHE 200 μg/mL; (5) K12-100 μg/mL BRHE; (6) K12-50 μg/mL BRHE; (7) K12-25 μg/mL BRHE. (**B**) Statistical analysis of the percentage of the cells engulfed K-12 particles. (**C**) Images of cell-engulfed K-2 particles by fluorescence microscopy with 100× magnification. Data are shown as the means ± SD of three replicates (n = 3). ** *p* < 0.01, compared to the control group.

**Figure 6:**
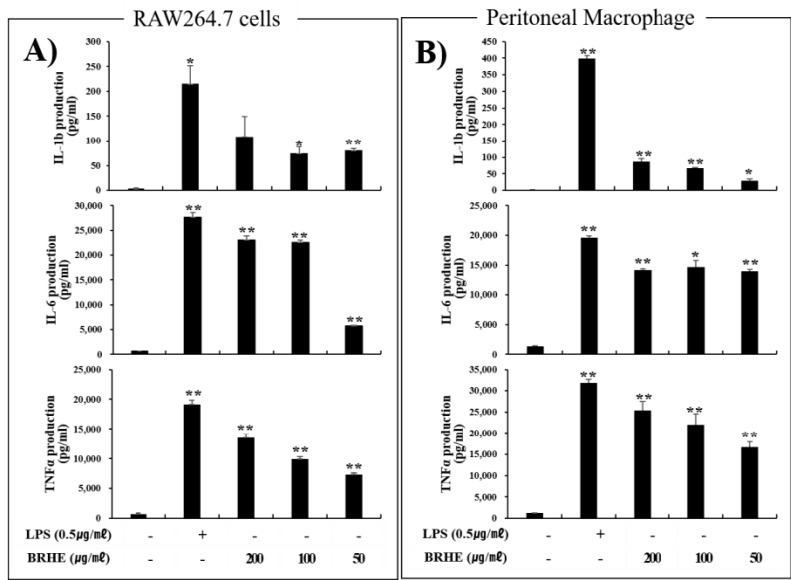
The stimulating effects of BRHE on RAW 264.7 cells and peritoneal macrophages. (**A**) RAW 264.7 cells and (**B**) peritoneal macrophage produced various cytokines, such as Ilβ, IL-6, and TNF-α. Data are shown as the means ± SD of three replicates (n = 3). * *p* < 0.05, ** *p* < 0.01, compared to the control group.

**Figure 7:**
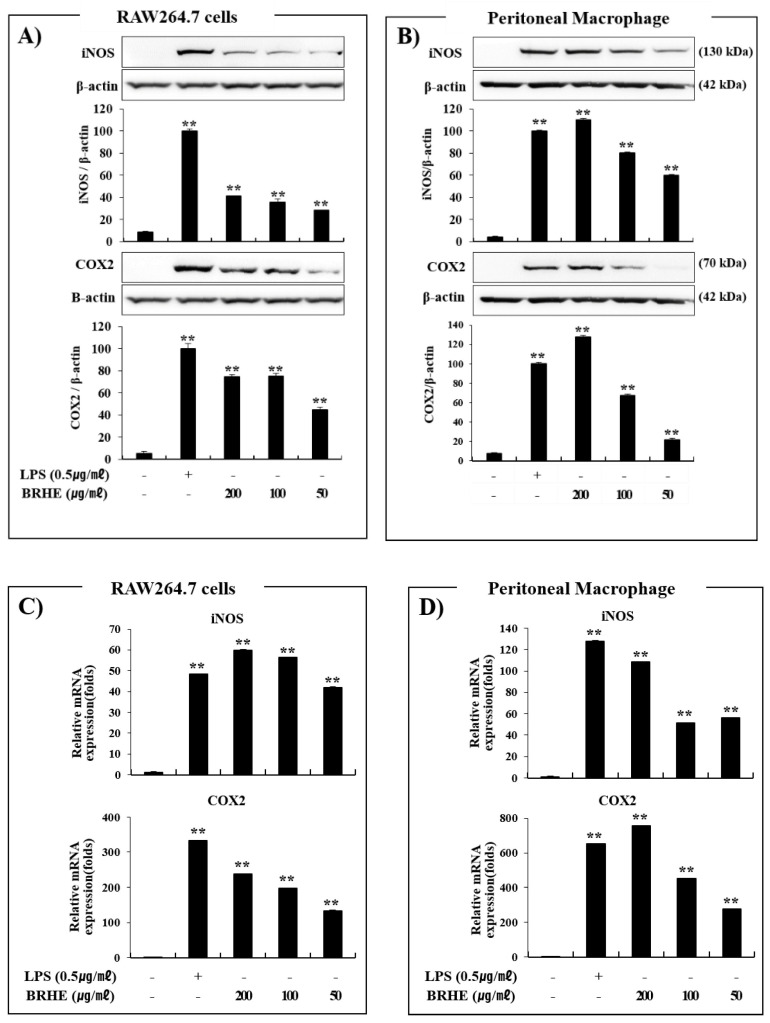
The effects of BRHE on the protein (**A**,**B**) and mRNA expression (**C**,**D**) of iNOS and COX-2 in RAW 264.7 cells and peritoneal macrophages. The cells were treated with BRHE (200, 100, or 50 μg/mL) or LPS (0.5 μg/mL) for 20 or 6 h. Representative pictures of Western blot or qPCR and quantitative analysis of blots. Each immunoreactive band was digitized and expressed as a ratio of β-actin levels. The data are expressed as mean ± SD of three independent experiments. ** *p* < 0.01, versus LPS treatment alone.

**Figure 8:**
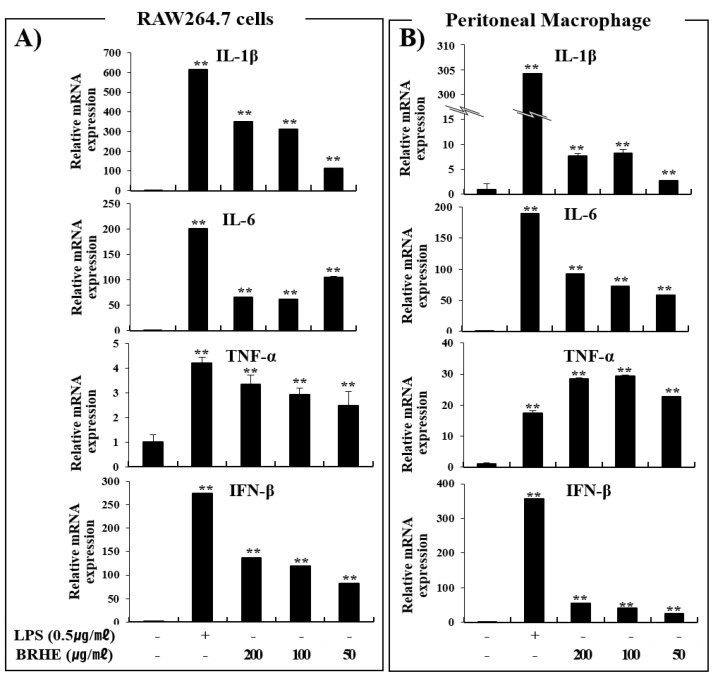
The effects of BRHE on IL-1β, IL-6, TNF-α and INFβ mRNA expression in RAW 264.7 macrophages (**A**) and peritoneal macrophage (**B**). The cells were pre-treated with BRHE (200, 100, or 50 μg/mL) or LPS (0.5 μg/mL) for 6 h. Cellular mRNA expression analysis was determined using qPCR. IL-1β, IL-6, TNF-α and INFβ expression levels were calculated after normalizing the signal against the β-actin gene. Data are expressed as mean ± SD of three independent experiments. ** *p* < 0.01 versus control.

**Figure 9:**
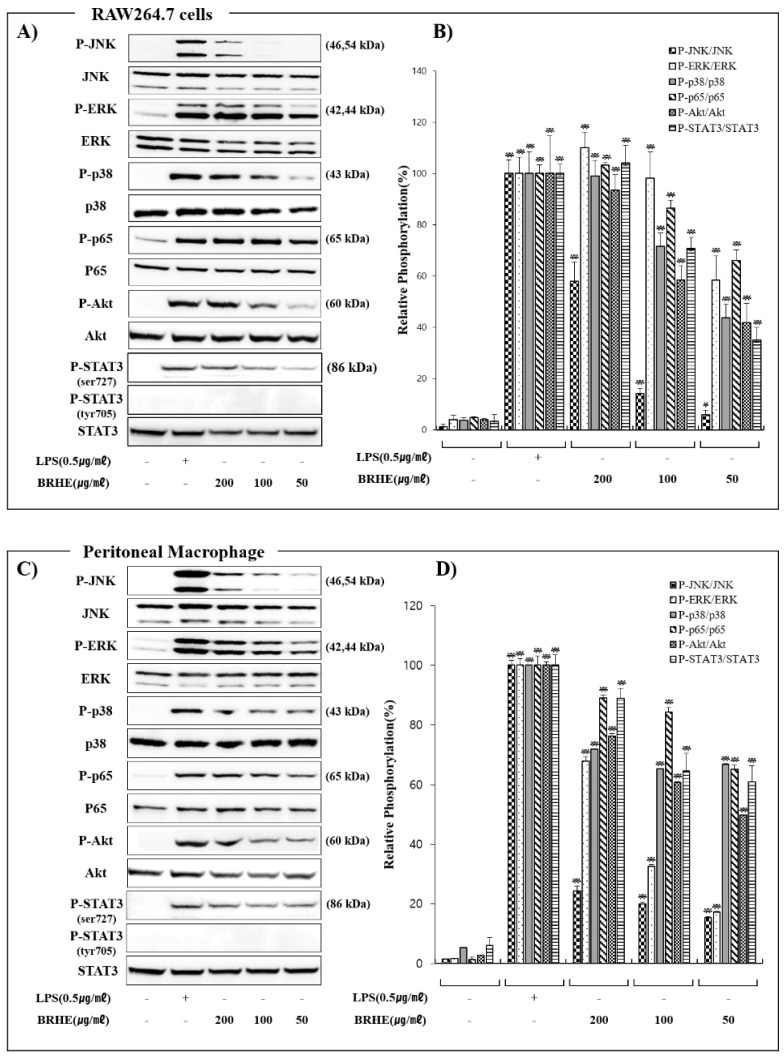
The effects of BRHE on JNK, ERK, P38, p65 (NFkB), Akt and STAT3 phosphorylation in RAW 264.7 cells (**A**) and peritoneal macrophage (**C**). The cells were treated with BRHE or LPS for 40 min. After treatment, the phosphorylated JNK, ERK P38, p65 (NFkB), Akt and STAT3 levels were analyzed by immunoblotting. Histogram represents quantification of BRHE-stimulated p-JNK, p-ERK p-JNK, p-p38, p65, p-Akt and p-STAT3 in RAW 264.7 (**B**) or peritoneal macrophage samples (**D**). All data of relative activity are expressed as a comparison with untreated cells. Data are expressed as mean ± SD of three independent experiments. * *p* < 0.05, ** *p* < 0.01 versus control.

**Figure 10:**
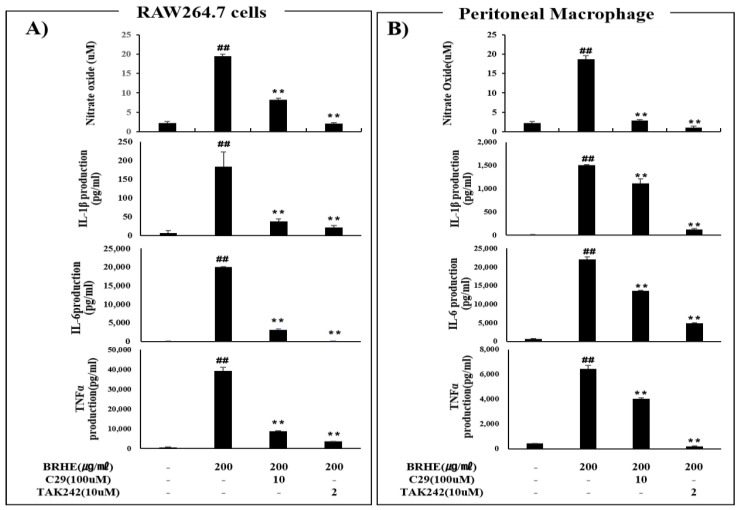
The effects of C29, TAK242 on BRHE induced NO, IL-1b, IL-6, and TNF-α production. The RAW 264.7 cells (**A**) and peritoneal macrophages (**B**) were pre-treated with C29 (TLR2 inhibitor), TAK242 (TLR4 inhibitor) for 30 min and then treated with BRHE (200 μg/mL) for 20 h. Representative pictures of Griss assay, ELISA, and quantitative analyses. Data are expressed as mean ± SD of three independent experiments. ## *p* < 0.01 versus control; ** *p* < 0.01 versus BRHE treatment alone.

**Figure 11:**
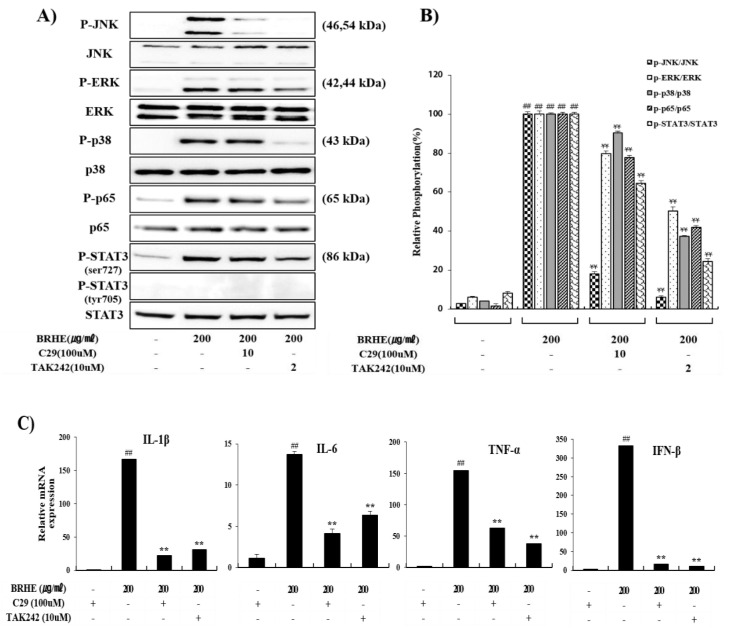
The effects of C29, TAK242 on BRHE induced phosphorylation (JNK, ERK, p65 (NFkB) and STAT3) and cytokine mRNA expression in RAW 264.7 cells. The RAW 264.7 cells were pre-treated with different C29 and TAK242 concentrations for 30 min and then treated with BRHE for 40 min. Representative pictures of Western blot (**A**), quantitative analyses (**B**) and cellular mRNA expression analysis using qPCR (**C**). Each immunoreactive band was digitized and expressed as a ratio of JNK, ERK, and p65 protein levels. Data are expressed as mean ± SD of three independent experiments. ## *p* < 0.01 versus control; ** *p* < 0.01 versus BRHE treatment alone.

**Figure 12:**
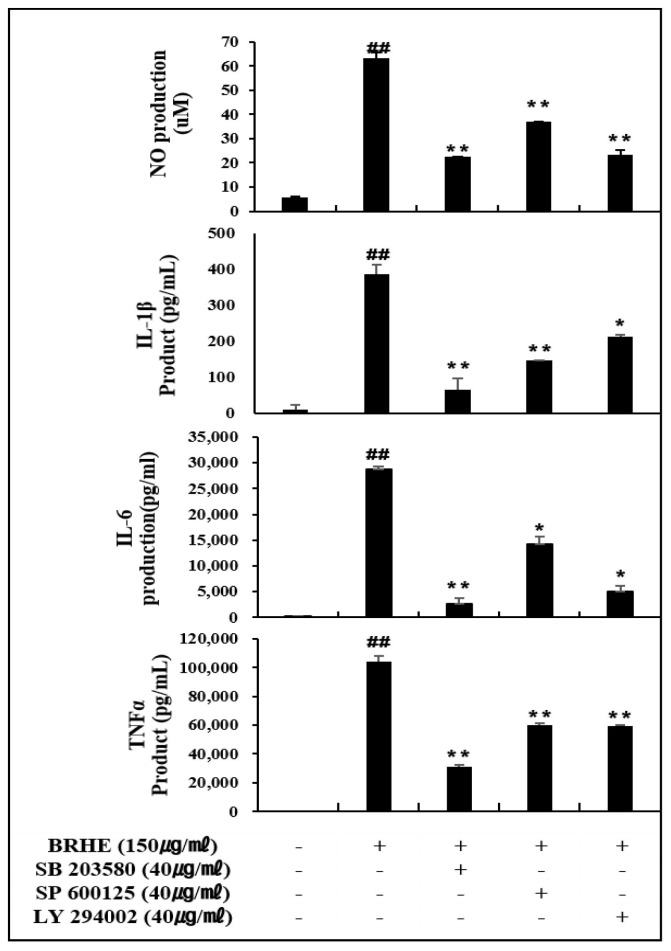
Effects of SB203580 (p35 inhibitor), SP600125 (JNK inhibitor) and LY294002 (PI3K inhibitor) on BRHE-induced NO, IL-1β, IL-6 and TNF-α production in RAW 264.7 cells. Data are presented as mean ± SD (n = 3). ## *p* < 0.01, * *p* < 0.05, ** *p* < 0.01 indicate statistical significant with BRHE alone.

We next investigated the effects of BRHE on the activation of macrophage phagocytosis. Furthermore, we conducted a flow cytometry assay and microscopy to verify phagocytosis. Thus, BRHE was added to final concentration of 200, 100, and 50 and 25 μg/mL increased phagocytosis activity by 28.73%, 25.31%, 20.06%, and 16.16%, respectively (Figure 5A,B). The flow cytometric plots of the phagocytic cell populations are shown in Figure 5A, and the mean fluorescent intensity (MFI) showed a trend that was consistent with the fluorescence microscopic analysis (Figure 5C). Specifically, as shown in Figure 5C, K-12 BioParticle-mediated phagocytic activity of RAW 264.7 cells was strongly enhanced by BRHE compared with the control. As shown in Figure 5B, the rate of phagocytosis of BRHE-treated cells significantly increased (48.49% vs. the control, 21.05%; (*p* < 0.01)). Furthermore, flow cytometric analysis reveals that enhanced phagocytosis levels by BRHE treatment were correlated with ROS production levels (Figure 4A,B). These results indicate BRHE significantly induced the promotion of macrophage phagocytosis.

### 2.6. BRHE Induces IL-1β, IL-6, and TNF-α Production by RAW 264.7 Cells and PMs

ELISA results showed that BRHE significantly increased (*p* < 0.01) the production of IL-1β, IL-6, and TNF-α by RAW 264.7 cells (Figure 6A) and PMs (Figure 6B), in contrast to the controls, in a concentration-dependent manner. Furthermore, the secretion of IL-1β, TNF-α and IL-6 was significantly increased (*p* < 0.01) after BRHE stimulation compared with that of the controls (Figure 6). Specifically, treated with BRHE concentrations of 200, 100 ug/mL in RAW 264.7 cells and PMs, BHRE promoted IL-1β, IL-6 and TNF-α production by 18~54, 11~35, and 17~20 folds, respectively, compared with that of the non-treated control cells. Expectedly, LPS (0.5 μg/mL) also highly induced the production of IL-1β, IL-6, and TNF-α in RAW 264.7 cells and PMs (Figure 6). These results indicate that BRHE is a potent stimulator for inflammatory cytokines, similar to that of the TLR4 ligand LPS acting on mouse macrophages (Figure 6A,B).

### 2.7. Effects of BRHE on iNOS and COX-2 Protein and mRNA Expression

To further demonstrate the immunostimulatory effect of BRHE, we determined the protein levels of iNOS and COX-2 using Western blotting. As shown in Figure 7A,B, BRHE treatment enhanced the expression of iNOS and COX-2 proteins in RAW 264.7 cells compared to untreated cells, of which effect was dependent on BRHE amount. 

We used LPS as a positive control to show the strong expression of iNOS and COX-2 compared with the controls. Further, the 200 ug/mL treatment of BRHE induced higher levels of iNOS and COX-2 protein in peritoneal macrophages than LPS (Figure 7B).

To investigate the effects of BRHE on the pro-inflammatory response at the transcriptional level, we determined the levels of iNOS and COX-2 mRNAs in BRHE-stimulated RAW 264.7 cells and PMs. As shown in Figure 7C,D, the levels of iNOS and COX-2 mRNA in both cell types were markedly increased following BRHE treatment in a concentration-dependent manner, and those in BRHE-stimulated PMs were highly enhanced than those in RAW 264.7 cells (Figure 7C,D). These results are consistent with the increase in NO production in both cell types (Figure 3C,D). Collectively, these results indicate that BRHE may possess significant immunomodulatory potential through stimulation of the expression of inflammatory cytokines and immunomodulatory factors. 

### 2.8. BRHE Enhances the Expression of mRNAs Encoding IL-1β, IL-6, TNF-α, and INFβ

As described above, BRHE treatment enhanced cytokine production in RAW 264.7 cells and PMs (Figure 6). Hence, we determined whether BRHE affected the levels of mRNA encoding IL-1β, IL-6, TNF-α, and INFβ in RAW 264.7 cells (Figure 8A) and PMs (Figure 8B). The levels of the mRNAs encoding IL-1β, IL-6, TNF-α, and INFβ in cells treated with BRHE were significantly increased (Figure 8). After treatment with 200 μg/mL concentration of BRH, we observed that the levels of IL-1β, IL-6, TNFα and INFβ mRNA were increased by 18~320, 60~90, 3.5~19, and 40~120 folds, respectively. Furthermore, qPCR analysis revealed that the mRNA expression levels of iNOS, COX-2, IL-1β, IL-6, and TNF-α correlated with their proteins levels (Figure 6 and Figure 7). These data suggest that BRHE-induced pro-inflammatory cytokine production increases the expression of IL-1β, IL-6, TNF-α, and INF-β mRNAs, thereby contributing to an immunomodulatory effect.

### 2.9. The Effects of BRHE on the Activation of MAPK, NFκB, PI3K/Akt, and STAT3 Signaling Pathways in RAW 264.7 Cells and PMs

We performed Western blot analyses to investigate the phosphorylation of ERK1/2, JNK, and p38 to determine whether the activation of the immunomodulatory response to BRHE was mediated through the MAPK signal transduction pathway. In these results, BRHE treatment of cells for 40 min significantly increased the activation of JNK, ERK, and p38 (not shown). We found that the BRHE significantly increased ERK1/2 JNK, and p38 phosphorylation in RAW 264.7 cells (Figure 9A,B) and PMs (Figure 9C,D) compared with the controls. Therefore, BRHE exerted a significant effect on the up-regulation of the phosphorylation of ERK1/2, JNK1/2, and p38, suggesting that these pathways were involved in the immunomodulatory activity of BRHE.

To identify the molecular mechanisms underlying the activation of macrophages by BRHE, we performed Western blot analysis to determine the effect of BRHE on the phosphorylation of the transcription factor NF-κB p65 that contributes to the regulation of the immune response [32]. Treatment of RAW 264.7 cells and PMs with BRHE or LPS induced the phosphorylation of NFkB p65 in a concentration-dependent manner (Figure 9). Similarly, we next evaluated the effects of BRHE on Akt, a downstream component of the PI3K pathway that mediates cell proliferation and survival. We found that BRHE elevated the phosphorylation levels of Akt in RAW 264.7 cells and PMs in a concentration-dependent manner (Figure 9). We next assessed whether BRHE induces both Ser727 and Tyr707 phosphorylation in STAT3 activation. When treated with LPS or BRHE in RAW 264.7 cells and peritoneal macrophages, we observed Ser727 phosphorylation in STAT3 after 40 min, but not Tyr707 phosphorylation, suggesting that BRHE may function as a modulator of STAT3 activity (Figure 9A,B). Thus, these results demonstrate that BRHE consistently modulated MAPK (ERK, JNK, and P38), NFkB, Akt, and STAT3 activity and may therefore serve as potential immunomodulatory agents.

### 2.10. The Effects of BRHE-Mediated Immunostimulation Are Mediated by TLR2 and TLR4

TLR2 and TLR4 are essential sensors and signal transducers for LPS, viruses, pathogens (proteins, peptidoglycans, lipoteichoic acid, zymosan, mannan), and various polysaccharides, which are strong immunostimulatory components in plants. Thus, we hypothesized that stimulation of TLR2 and TLR4 with BRHE alters the levels of NO and cytokines, leading to immunostimulation. Therefore, we determined whether TLR2 and TLR4 were involved in the activation of murine macrophages by BRHE treatment. After TLR2 and TLR4 were inhibited with C29 and TAK-242, respectively, RAW 264.7 cells and PMs were treated with BRHE, and the production of immunomodulators, such as NO, IL-1, IL-6, and TNF-α, were measured (Figure 10). In contrast to the significant increase in NO product induced by BRHE in RAW264 cells, NO products were decreased in BRHE-stimulated cells after treatment with the TLR2 inhibitor (C29) and TLR4inhibitor (TAK242). As shown in Figure 10A,B, these TLR inhibitors reduced the production of immunomodulators, such as IL-1β, IL-6, and TNF-α, in cells treated with BRHE compared with cells treated with BRHE alone. However, the inhibition of TLR4 by TAK242 was strongly blocked compared with TLR2 by C29 in RAW 264.7 cells (Figure 10A) and PMs (Figure 10B). 

We investigated whether the attenuation of NO, IL-1β, IL-6, TNF-α, and INF-β levels in the presence of TLR inhibitors was influenced the activities of MAPKs (JNK, ERK, p38), NFkB (p65), Akt, and STAT3 (Ser727). We found that the levels of phosphorylated MAPKs (JNK, ERK,p38), NFkB (p65), and STAT3 (Ser727) in RAW 264.7 cells were significantly decreased compared with those of cells treated with BRHE alone (Figure 11A,B). Therefore, we performed an investigation into the effect of TLR2/4 inhibitors on BRHE-mediated cytokine mRNA expression, compared with that of control cells. As expected, C29 and TAK242 inhibited the BREH-mediated immunomodulator mRNA expression, such as IL-1β, IL-6, TNF-α and INF-β (Figure 11C). Overall, these results indicate that BRHE can activate macrophages through the TLR2/4 signal pathway, and that the affinity between BRHE and TLR linkage is much higher on TLR4 than TLR2 response signals.

### 2.11. Effects of BRHE-Mediated Signaling by MAPK and Akt Inhibitors

To investigate whether the MAPK and Akt signaling pathways were involved in macrophage activation by BRHE, RAW 264.7 cells were treated with BRHE to assess the levels of NO in cells co-treated with p35, JNK and Akt inhibitors SB203580, SP600125, and Ly294002, respectively. As shown in Figure 12, the NO level was highly increased after treatment with BRHE, but, RAW 264.7 cells treated with the p35, JNK and Akt inhibitors were attenuated by 64.6%, 41.6% and 63.1%, respectively (Figure 12). These results suggest that the phosphorylation of MAPK and Akt are involved in the stimulation of macrophages by BRHE. To provide further evidence that the MAPK and Akt pathways were involved in macrophage activation by BRHE, the p35, JNK and Akt inhibitors were added to macrophages treated with BRHE, and the levels of inhibition of IL-1β, IL-6, and TNFα secretion were determined. As shown in Figure 12, RAW 264.7 cells treated with BRHE secreted high levels of IL-1β, IL-6, and TNF-α; however, the inhibitors SB203580, SP 600125, and Ly294002 suppressed the induction of IL-1β, IL-6, and TNF-α by BRHE (Figure 12). These results indicate that the MAPK and Akt signaling pathways may be involved in BRHE-induced production of immunomodulators in macrophages. 

Therefore, MAPK and Akt serve as mediators that contribute to the production of immunomodulators in BRHE-treated RAW 264.7 cells. Thus, MAPK and Akt activation in TLR2 and TLR4 signal pathways stimulated with BRHE are one component important in the production of immunomodulators on macrophages, which is supported by the observation that natural products stimulate the TLR2/4 signaling pathway, and in turn MAPK/Akt phosphorylation in macrophages [29,30,32]. 

## 3. Discussion

The black radish is a popular food that is mainly consumed in Japan, Korea, and China [3,33]. Radish roots are primarily used as a food and food supplement. The beneficial effects of its roots, sprouts, seeds, and leaves include detoxification in HepG2 cells [34] as well as antioxidant [33], anti-inflammatory [35], gastroprotective [36], antifibrotic [37], anti-obesity [38], and antidiabetic [22] properties. These beneficial effects are often attributed to it containing sulfur compounds, phenolic compounds, glucosinolates, and flavonoids [19,39]. Among the parts of the black radish, the leaves and roots are used to treat respiratory infections, fever, and inflammatory diseases. Further, the roots of the black radish are used as a food source, and when fermented attenuate NAFLD and confers a hepatoprotective effect via the inhibition of TGFβ/collagen type 1 synthesis [40]. However, studies on the immunomodulatory activity of BRHE are insufficient.

Macrophages play an important role in the host’s defense system against diverse pathogenic infections. For example, polysaccharides isolated from natural sources (microorganisms, including *Lactobacillus*, algae, and mushrooms, and higher plants) enhance the protective activities of macrophages against pathogenic microorganisms and tumor cells by the secretion of ROS, NO, and cytokines [40,41,42,43]. Here, we identified novel hot water-soluble polysaccharides from BRHE with molecular masses in the range 185~9.08 × 10^5^ Da. Monosaccharide analysis revealed that BRHE mainly comprised glucose, rhamnose, arabinose, and galactose (84.5, 5.0, 2.0, and 2.7%, respectively). To the best of our knowledge, we have demonstrated for the first time that BRHE comprises a population of immunostimulatory polysaccharides, indicating that they interact with the immune system to strengthen host reaction-specific processes.

Immunomodulatory polysaccharides represent an immune system that interacts with pattern recognition receptor (PRR) reaction-specific processes such as those mediated by the TLRs–MyD88–MAPK (ERK, JUN, p38) or NFκB pathways [13,44]. Studies regarding immunomodulatory polysaccharides focus mainly on glucans, pectic polysaccharides, and arabinogalactans [45]. The immunostimulatory activities of these polysaccharides are influenced by their source, molecular mass, monosaccharides, glycosidic linkages, functional groups, and branching characteristics [45]. For example, Zheng et al. [46] reported that rhamnogalacturonan-1 (RG-I) pectin achieved more potent induction of lymphocyte proliferation compared with homogalacturonan (HG) pectin. Arabinogalactans (AG) is an effective immunomodulator, and the AG chain contributes to promoting NO production and lymphocyte proliferation. Moreover, immunostimulatory polysaccharides are ubiquitous in diverse fruits and vegetables, such as okra [30], pumpkin [47,48], and citrus fruit [41]. Together with the present study, these findings support the conclusion that polysaccharides contribute to the regulation of the immune system. Studies of the structural characteristics of BRHE polysaccharides are required to identify glycosidic linkages, functional groups, and branching characteristics.

Macrophage phagocytosis and ROS production induced by BRHE is accepted as an important step in immunomodulatory activity. Phagocytosis, which is the first step in the response of an activated macrophage to invading pathogens or microorganisms, contributes to the innate immune response. Phagocytic macrophages produce ROS or NO. Moreover, activated macrophages secrete more cytokines or immunomodulators, such as IL-1, IL-6, and TNF-α [12]. These secreted immunomodulators directly destroy microbial pathogens and cancer cells [31,49,50]. Thus, macrophage activation serves as an important target for strengthening the human innate immune system [51]. Further, the innate immune response of activated macrophages contributes to the strengthening of the adaptive immune response, which is mediated by vascular relaxation, vasodilatation as well as the activation of natural killer cells, T cells, and B cells [52,53].

Here, we found that BRHE promoted the generation of NO and ROS and cytokine secretion by RAW 264.7 macrophages and PMs and enhanced the phagocytic uptake capacity of RAW 264.7 cells (Figure 3, Figure 4, Figure 5 and Figure 6). These results indicate that BRHE represents a new immunostimulant for macrophages. Further, iNOS and COX-2 proteins and mRNA expression levels in BRHE-treated RAW 264.7 cells and PMs were significantly increased (Figure 7). This study provides compelling evidence that BRHE induces NO production by enhancing iNOS and COX-2 expression to achieve an immunomodulatory effect.

The molecules of the MAPK family of serine/threonine-specific protein kinases, which includes p38, JNK, and ERK, are required to facilitate the production of pro-inflammatory mediators [29,32]. For example, recent studies have shown that water extracts from *Artemisia princeps* [54], *Prunella vulgaris* [55], and *Mori fructus* [56] trigger phosphorylation of the components of the MAPK signaling pathway, resulting in the release of cytokines. Moreover, polysaccharides derived from *Hibiscus sabdariffa* Linn [57], *Angelicae dahuricae* [58], and *Cordyceps militaries* [59] activate macrophages to produce cytokines and NO through the phosphorylation of MAPK, NFκB, and Akt.

Consistent with the results of these previous studies, we show here that BRHE activates macrophages through the MAPK (ERK, JNK, p38), NF-κB, and PI3K/Akt signaling pathways, as indicated by the changes in the phosphorylation of MAPK, NF-κB, and Akt as well as increases the levels of NO or cytokines (IL-1β, IL-6 and TNF-α) in RAW 264.7 cells (Figure 9A) and PMs (Figure 9B). These results suggest that black radish polysaccharides mediate the activation of the MAPK, NF-κB, and PI3K/Akt signaling pathways.

TLRs serve as PRRs for macrophages. TLR2 and TLR4 are transmembrane receptors that transmit plant polysaccharide signals to intracellular components of signal transduction pathways and play important roles in the immune system [60]. Moreover, TLR2 and TLR4 mediate polysaccharide-induced cytokine or NO production [61,62] and trigger signal transduction through MAPKs, including ERK, p38, and JNK. Further, MAPK, NFκB, and PI3K/Akt signaling mediates TLR2/4-dependent macrophage activation in response to polysaccharides, including *Sageretia thea* [63], *Physalis alkekengi* [64], fresh longan [65], and *Citrus unshiu* [66]. Thus, we employed specific inhibitors of TLR2 and TLR4 to determine whether TLRs were involved in the regulation of the production of NO, cytokines (IL-1β, IL-6, and TNF-α) and MAPK phosphorylation, including NFkB, STAT3. Our results indicate that the TLR2 and TLR4 inhibitors C29 and TAK242 significantly inhibited the production of NO, IL-1β, IL-6, and TNF-α (Figure 10A,B) as well as the phosphorylation of MAPKs (ERK, JNK), P65 and STAT3 level (Figure 11A,B). Further, C29 and TAK242 significantly inhibited the expression of mRNAs encoding IL-1β, TNF, and IL-6 in RAW 264.7 cells (Figure 11C). As shown in Figure 11, TLR4 inhibition by TAK242 blocked the activation of ERK1/2, JNK, NFkB, and STAT3 stronger than TLR2 inhibition by C29. Furthermore, we confirmed that BRHE induced phosphorylation of ERK, JNK, p38, NFkB and STAT3 in RAW 264.7 cells by Western blotting (Figure 11A).

**Figure 13:**
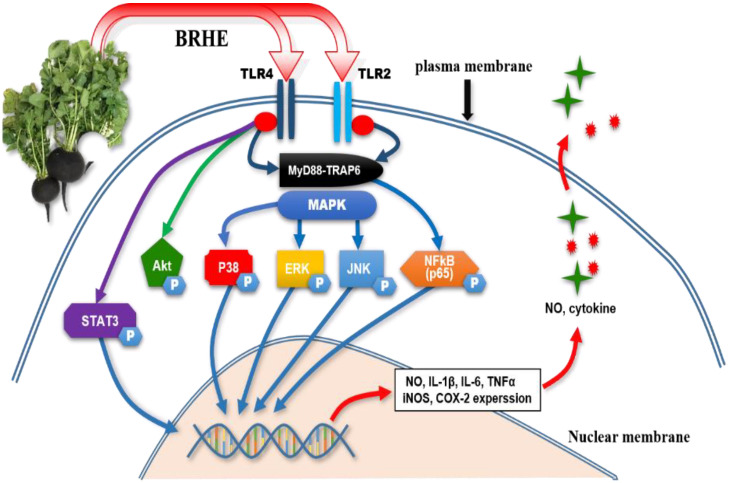
Schematic illustration of the immunomodulatory effects of BRHE in a mouse macrophage. BRHE may be beneficial in triggering immunomodulatory effects, which may be mediated by TLR2/4–MAPK–NFkβ–Akt–STAT3 signal transduction pathway activation. The arrows represent activation, while the T-shaped arrows indicate inhibition. TLR2/4, toll-like receptor2/4; BRHE, black radish hot extract; MAPK, mitogen-activated protein kinase; MyD88, myeloid differentiation primer response 88; TRAF6, tumor necrosis factor receptor-associated factor 6; Akt, protein kinase B; ERK, extracellular signal regulated protein; JNK, c-Jun NH-2 terminal protein; NFkβ, nuclear factor kappaβ; STAT3, signal transduction activator of transcription 3; iNOS, inducible nitric oxide synthase; NO, nitric oxide; COX-2, cyclooxygenase-2; TLR4, toll-like receptor 4; TNF-α, tumor necrosis factor alpha; IL-1β, interleukin 1β.

As shown in Figure 12, the p35 inhibitor (SB203580), JNK inhibitor (SP600125) and Akt inhibitor (Ly294002) were significantly suppressed in BRHE-induced NO, IL-1β, IL-6 and TNF-α release in RAW 264.7 cells. These results demonstrated that BRHE plays a role in the MAPK and Akt pathways involved in macrophage activation. These results suggest that BRHE triggers signaling through the MAPK signaling pathway through TLR2 and TLR4, leading to macrophage activation. Moreover, pre-treatment with TLR2 and TLR4 inhibitors partially inhibited the production of NO, suggesting that TLR2 and TLR4 are the two main receptors required for BRHE-mediated immunomodulatory activity in RAW 264.7 cells (Figure 13). Thus, our data demonstrate that not only TLR4 signaling, but also TLR2 appears to be an essential accessory for immune responses through BRHE signaling. The function of TLR2 and TLR4 in response to TLR ligands could be associated with each other by BRHE. These results can help us understand the unique role of BRHE in the macrophage response to TLR ligands. 

In conclusion, we isolated and characterized a novel water-soluble heteropolysaccharide, termed BRHE, from the black radish root and evaluated its immunomodulatory activities in mouse RAW 264.7 cells and PMs. The BRHE exhibited significant immunomodulatory activities by up-regulating the secretion of TNF-α, IL-6, ROS, and NO. Further, the MAPK, PI3K/Akt, NF-κB and STAT3 signaling pathways from macrophages, involved in BRHE-induced macrophage activation. Moreover, TLR2 and TLR4 may play an important role in the activation of macrophages via the MAPK (ERK, JNK, and p38) signaling pathway triggered by BRHE (Figure 13). These results indicate that BRHE may contribute to the immunomodulatory effect of signaling through the TLR2-4–MAPK–NFkB–Akt–STAT3 pathway in macrophages (Figure 13). Together, these results provide a better understanding of the molecular mechanisms of the activation of mouse macrophages induced by BRHE. Thus, BRHE shows potential for its application as a complementary dietary bioactive ingredient in healthy foods as well as an immunomodulator.

## 4. Materials and Methods

### 4.1. Chemicals and Kits

DMEM, trypsin, and penicillin–streptomycin were purchased from Gibco (Grand Island, NY, USA). Fetal bovine serum (FBS) was purchased from Gibco, and 3-(4,5-dimethy-2-thiazolyl)-2,5-diphenyl-2H-tetrazolium bromide (MTT), DCAFD, and LPS were obtained from Sigma–Aldrich korea (Gangnam-gu, Seoul, Korea). TAK242 (TLR4 inhibitor) and C29 (TLR2 inhibitor) were purchased from Sigma–Aldrich korea (Gangnam-gu, Seoul, Korea) and Biovision (Waltham, MA, USA), respectively. Enzyme-linked immunosorbent assay (ELISA) kits for mouse IL-6, IL-1β, and TNF-α were purchased from R&D Systems (Minneapolis, MN, USA). A Vybrant phagocytosis assay kit was purchased from Invitrogen (Waltham, MA, USA), NucleoSpin RNA reagent was obtained from Macherey–Nagel (Dueren, Germany), and the ReverTra Ace-α First Strand cDNA Synthesis Kit was procured from Toyobo korea (Gangnam-gu, Seoul, Korea). FastStart Universal SYBR Green Master (Bio-Rad) was purchased from Bio-Rad (Hercules, CA, USA), and C29 and TAK242 were obtained from MediChemExpress (Woodridge, IL, USA) and Sigma–Aldrich Korea (Gangnam-gu, Seoul, Korea), respectively. SB203584 (ERK inhibitor), SP600125 (JNK inhibitor), and LY294002 (Akt inhibitor) were purchased from Sigma–Aldrich korea (Gangnam-gu, Seoul, Korea) and Calbiochem (Darmstadt, Germany). Antibodies against COX-2, β-actin, and iNOS were purchased from Calbiochem (Darmstadt, Germany); and antibodies against STAT3, 18hioglyc-STAT3 (Tyr 703, Ser727), 18hioglyc-ERK, ERK, 18hioglyc-JNK, JNK, 18hioglyc-JUN, JUN, 18hioglyc-p38, p38, 18hioglyc-p65, and p65 were purchased from Cell Signaling Technology (Beverly, MA, USA). All other reagents were of analytical grade.

### 4.2. Preparation of BRHE

Black radishes were obtained from Sungsan Ilchubong Nonghyup, whose planting facility is located in Jeju Province, Korea. Black radish roots (5000 g) were cut into thin slices, dried at 45 °C, and extracted three times at 80 °C–90 °C for 6 h with distilled water. The extracts were filtered through Whatman no. 3 filter paper and then concentrated 5-fold at 44 °C using a Buchi R-210 rotary evaporator. The BRHE was dried in a freeze-drying oven for 3 days and then stored at −20 °C.

### 4.3. Determination of the Molecular Weight of BRHE

For molecular weight measurement, the extracted black radish roots from hot water was analyzed by high-performance size-exclusion chromatography (HPSEC) and refractive index (RI) systems (HPLC-Agilent 1100, Santa Clara, CA, USA). The BRHE sample (100 μL) was injected into Shodex SB-804 HQ and Shodex SB-802 5HQ OHPak columns (8.0 mm ID × 300 mm, Showa, Denko, Japan) after being passed through nylon filter (5.0 μm, Thermo Scientific, Inc., Waltham, MA, USA). The eluent was water at a flow rate of 0.6 mL/min. Molecular mass standards, Maltooligosaccharides (G1–G7) and Shodex standard P-82 (P5, P10, P20, P100, P200, and P400) were used. The molecular mass of the BRHE was measured using a GPC ELEOS System (Wyatt Technologies Inc., Goleta, CA, USA).

### 4.4. Analyses of Monosaccharide and Galacturonic acid Compositions

The analyses for carbohydrate, protein, fatty acid, and ash in BRHE were conducted according to a previously published method [67]. Total sugar content was measured using the phenol-sulfuric acid method with D-glucose (Sigma–Aldrich) as the standard. To analyze the compositions of monosaccharides and galacturonic acid in BRHE solutions (1%, *w*/*v*), the vacuumed Reacti-Vial™ Small Glass Reaction Vials (Thermo Fisher Scientific, Rockford, IL, USA) were hydrolyzed with 2 M trifluoracetic acid (TFA; Sigma-Aldrich Chemical Co.) at 120 °C for 2 h in a heating module (Thermo Fisher Scientific, Rockford, IL, USA). The amount of individual monosaccharides and galacturonic acid were examined by high-performance anion-exchange chromatography (HPAEC, Thermo Scientific, Sunnyvale, CA, USA) with a pulsed amperometric detector (PAD, Thermo Scientific, Sunnyvale, CA, USA). Chromatographic separation of the monosaccharides was achieved isocratically in a CarboPacTM PA-1 column (Thermo Scientific, Sunnyvale, CA, USA) with 20~250 mM NaOH for 27 min at a flow rate of 0.8 mL/min. 

### 4.5. Cell Culture

The RAW 264.7 cell line was purchased from the Korean Cell Line Bank (Jongno-gu, Seoul, Korea). The cells were cultured in DMEM containing 2 mM glutamine, 4.5 g/L glucose, 100 mg/L sodium pyruvate (Gibco, PA, USA), penicillin (100 units/mL), streptomycin (100 μg/mL), and 10% FBS. The cells were cultured at 37 °C in a humidified incubator in an atmosphere containing 5% CO_2_, and 5 × 10^5^ cells were added to the wells of a 6-well cell culture plate, incubated for 16 h, and then treated with LPS (1 μg/mL) with or without BRHE (200, 100, 50, and 25 μg/mL) for 6–24 h.

### 4.6. Preparation of Mouse Peritoneal Macrophages

Mouse peritoneal macrophages were collected 3 days after C57BL/6 male mice were injected with 1 mL of 4% 19hioglycolate medium. Peritoneal macrophages (PMs) were isolated from 6–8-week-old pathogen-free mice. Briefly, mice were intraperitoneally injected with 1 mL of 4% 19hioglycolate. Four days later, cells were harvested using peritoneal lavage with cold PBS. Cells were recovered via centrifugation and cultured in RPMI 1640 (2 mM L-glutamine, antibiotics) supplemented with 5% FBS and then were seeded into the wells of 12- or 6-well plates (1 × 10^6^ cells/well). The cells were allowed to adhere for 2 h, and then the medium was changed to remove non-adherent cells. After 24 h, the medium was replaced with a new complete medium before treatment. Each sample (20 μL) was added to a flat bottom 96-well tissue culture plate (Iwaki Science Department, Asahi Glass Corporation, Chiyoda-ku, Tokyo, Japan) at 10^5^ cells/well in 200 μL of RPMI 1640 medium. The medium was supplemented with L-glutamine, 25 mM HEPES, 10% FBS, and 100 μg/mL each of penicillin and streptomycin. The cells were incubated at 37 °C for 24 h.

### 4.7. Cell Viability Assay

Cell viability was measured using an MTT assay. Cells (1 × 10^4^ cells/well) were added to the wells of a 96-well cell culture plate, incubated for 24 h, and treated with different concentrations of BRHE (50, 100, 200, and 400 μg/mL) for 20 h. The cells were treated with LPS as the positive control. MTT was dissolved in phosphate-buffered saline to 2 mg/mL. After incubation, the cells were treated with 50 μL of MTT solution for 2 h, the solution was removed, and dimethyl sulfoxide (DMSO) was added to dissolve the formazan crystals. The concentration of dissolved formazan was measured at 540 nm using a microplate reader (BioTek, Winooski, VT, USA).

### 4.8. NO Assay and Treatment of TLR2,4 Inhibitors

The NO assay was performed as previously described with slight modifications [32]. After incubating RAW 264.7 cells (2 × 10^5^ cells) with LPS (0.5 μg/mL) for 20 h, the nitrite (NO_2_^−^) level in the culture medium was measured as a surrogate for NO production. The amount of NO_2_^−^, a stable metabolite of NO, was measured using Griess reagent (1% sulfanilamide and 0.1% N-1[naphthyl]ethylenediamine dihydrochloride in 2.5% phosphoric acid). Cell culture medium (100 μL) was mixed with 100 μL of Griess reagent, incubated at room temperature for 10 min, and the absorbance was measured at 540 nm using a microplate reader. Fresh culture medium was used as a blank. The NO_2_^−^ level was determined from a standard curve for NO_2_^−^. To identify the effects of the TLR2 and TLR4 on cytokine production, the RAW 264.7 cells were treated with or without the TRL2 inhibitor C28 (100 μM) or the TLR4 inhibitor TAK242 (10 μM) for 30 min. Then, BRHE (200 μg/mL) and LPS (0.5 μg/mL) were added to the RAW 264.7 cells. NO production and cytokine release in the culture medium was measured using Griess reagent and ELISA, as described above.

### 4.9. ROS Production Assay

ROS production by RAW 264.7 cells was measured using the fluorescent probe DCFH-DA. The RAW 264.7 cells (5 × 10^5^ cells/well) were added to glass-bottom cell culture dishes overnight and then incubated with BRHE (200, 100, 50, and 25 μg/mL) or LPS (0.5 μg/mL). LPS (0.5 μg/mL) served as a positive control. After 24 h, the culture medium was removed, and the cells were incubated with 10 μM DCFH-DA for 30 min at 37 °C. DCFH-DA was then removed, the cells were washed three times with DMEM without FBS, and fluorescence intensity was quantified using a flow cytometer (BD Bioscience, Franklin Lakes, NJ, USA). 

### 4.10. Phagocytosis Assay

To determine the activating effect of BRHE on RAW 264.7 cells and to determine the concentrations of BRHE used in subsequent experiments, macrophage phagocytic activity was tested using a Vybrant phagocytosis assay kit (V-6694). Briefly, RAW 264.7 cells were added to the wells of a 24-well plate (5 × 10^5^ cells/well) and incubated for 18 h, after which the supernatant was replaced with DMEM, LPS (1 μg/mL), or BRHE (200, 100, 50 and 25 μg/mL); and each treatment was repeated three times. After 18 h, the supernatant was removed, and the cells were treated with heat-killed fluorescein-labeled *Escherichia coli* K-12 BioParticles for 2 h. After removing the supernatant, trypan blue reagent was added to the 24-well plate for 1 min, and the cells were washed with PBS and immediately fixed with 1% formaldehyde for 30 min. The cell suspension was subjected to fluorescence-activated cell sorting using an FACSCalibur (BD Biosciences) (FL-1 channel, 564–606 nm).

### 4.11. Cytokine Assays

The BRHE was diluted with DMEM before use. The cells were treated for 24 h with LPS (0.5 μg/mL) to induce cytokine production. The concentrations of IL-1, IL-6, and TNF-α in culture medium supernatants were measured using an ELISA (R&D Systems, Minneapolis, MN, USA) as per the manufacturer’s instructions. The cells treated with only LPS (0.5 μg/mL) or those cultured in DMEM medium served as positive and negative controls, respectively.

### 4.12. Effects of Inhibitors of MAPK, and Akt

RAW 264.7 cells were first treated with specific inhibitors (ERK inhibitor 40 μM PD98059, JNK inhibitor 40 μM SP600125, P38 inhibitor 40 μM SB203580, and Akt inhibitor 40 μM LY294002 for 1 h and then incubated with BRHE (200 μg/mL) for 24 h. Cells or cell culture media was harvested and analyzed using the Griess assay and ELISA kit described above.

### 4.13. Real-Time Quantitative PCR (qPCR) Analysis

RAW 264.7 cells were collected from 6-well plates, and total RNA was prepared using the NucleoSpin RNA reagent (Macherey–Nagel, Dueren, Germany) according to the manufacturer’s instructions. A First Strand cDNA Synthesis Kit (Toyobo, Kyobashi, Tokyo, Japan) was used to generate cDNAs. The qPCR assays were performed using a premix (5X HOT FIREPol EvaGreen qPCR Supermix; Solis BioDyne, Tartu Science Park, Estonia) and a Bioer thermal cycler (Hangzhou, China) with primers (Bioneer, Daejeon, Korea) specific for the mRNAs encoding IL-1β, IL-6, TNF-α, COX-2, and iNOS (Table 2).

### 4.14. Western Blot Analysis

RAW 264.7 cells were lysed in RIPA buffer, and supernatant samples (40 μg protein) were loaded into the lanes of a 10% SDS–polyacrylamide gel, electrophoresed, and then electrophoretically blotted onto a nitrocellulose membrane (Schleicher and Schuell, Keene, NH, USA). The membrane was then incubated with antibodies against iNOS (1:1000 dilution), COX-2 (1:1000), p-STAT3 (1:1000), p-JNK (1:1000), p-ERK (1:1000), p-NFκB (p65) (1:1000), p-P38 (1:1000), JNK (1:1000), ERK (1:1000), NFκB (p65) (1:1000), or P38 (1:1000) for 2 h. The immunocomplexes were detected using a chemiluminescent substrate (Miracle-Star; iNtRON Biotech, Gyeonggi, Korea) according to the manufacturer’s instructions. After imaging, the membranes were stripped and probed again with the anti-β-actin antibody. The optical density (OD/mm^2^) of each band was measured, and the band density relative to that of the β-actin band was compared using ImageJ software. The stripped membranes were then incubated with antibodies against COX-2, iNOS, ERK, JNK, p38, or NFkBp65 overnight at 4 °C. The membranes were washed three times for 10 min between each step, incubated with horseradish peroxidase-conjugated secondary antibodies for 30 min at room temperature, and then washed again.

### 4.15. Statistical Analysis

The data are presented as the mean ± standard error. Statistical analysis was performed using the two-tailed Student’s *t* test or ANOVA for comparisons between or among two or multiple groups, respectively, and *p* < 0.05 indicates a significant difference.

## Figures and Tables

**Table 1:** The yield and compositions of BRHE obtained from black radish root.

Chemical Composition	BRHE (%, W/W)
Extract yield Total protein	38.7 9.6
Carbohydrate Total polysaccharide	71.3 11.7
Total sugar	46.6
Acidic sugar Fatty acid Ash content Moisture	2.5 0.1 12.4 6.6
**Neutral and Acid** **Sugar**	**Sugar Composition in BRHE (%)**
Fucose	0.22
Rhamnose	2.10
Arabinose	5.10
Galactose	5.28
Glucose	84.58
Xylose Galacturonic acid	2.71 13

**Table 2:** Primer sequences used in real-time quantitative polymerase chain reaction (qRT-PCR) analysis.

Target Gene	Forward Primer Sequence (5′ → 3′)	Reverse Primer Sequence (5′ → 3′)
iNOS	AACATCAGGTCGGCCATCACT	CCAGAGGCAGCACATCAAAGC
COX-2	GCAAATCCTTGCTGTTCCAAT	GGAGAAGGCTTCCCAGCTTTTG
IL-1β	CGTTCCCATTAGACAACTGCA	GGTATAGATTCTTTCCTTTGAGGC
IL-6	ACGGCCTTCCCTACTTC	TTCCACGATTTCCCAGA
TNF-α	CAAGGGACTAGCCAGGAG	TGCCTCTTCTGCCAGTTC
IFN-β	TCCAAGAAAGGACGAACATTC	TGCGGACATCTCCCACGTCAA
β-actin	CATCCTGCGTCTGGACCTGG	TAATGTCACGCACGATTTCC

## Data Availability

Data is contained within the article.
